# Preparation of Microspheres and Monolithic Microporous Carbons from the Pyrolysis of Template-Free Hyper-Crosslinked Oligosaccharides Polymer

**DOI:** 10.3390/molecules25133034

**Published:** 2020-07-02

**Authors:** Anastasia Anceschi, Andrea Binello, Fabrizio Caldera, Francesco Trotta, Marco Zanetti

**Affiliations:** 1 Department of Chemistry, NIS and INSTM Reference Centres, University of Torino, Via P. Giuria 7, 10125 Torino, Italy; anastasiaandrea.anceschi@unito.it (A.A.); andrea.binello@unito.it (A.B.); fabrizio.caldera@unito.it (F.C.); francesco.trotta@unito.it (F.T.); 2 ICxT Centre, University of Torino, Lungo Dora Siena 100, 10153 Torino, Italy

**Keywords:** nanosponges, carbon monolith, microporous carbon, hyper-crosslinked oligosaccharides, cyclodextrin, amylose

## Abstract

Carbon-based materials with different morphologies have special properties suitable for application in adsorption, catalysis, energy storage, and so on. Carbon spheres and carbon monoliths are also nanostructured materials showing promising results. However, the preparation of these materials often require the use of a template, which aggravates their costs, making the operations for their removal complex. In this work, hollow carbon microspheres and carbon monolith were successfully prepared via carbonization of hyper-crosslinked polymer based on either cyclodextrins or amylose, in a template-free way. The carbons obtained are of the microporous type, showing a surface area up to 610 m^2^/g, and a narrow pore distribution, typically between 5 and 15 Å.

## 1. Introduction

Thanks to the ability of carbon to take on different configurations, it has been possible to obtain a wide range of materials with an immensely different variety of structures and morphologies. Carbon materials, in particular porous ones, are generally characterized by their physical characteristics, which allow them to be used in many applications [1], such as food processing [2], chemical and pharma fields [3], petroleum industries [4] in water/air treatment [5], catalysis support [6], and fuel cell [7]. The possibility to use them in this wide range of applications certainly depends on their specific surface areas and porosity, but also on the ability to take the shape required for each specific application. For this reason, porous carbon materials are industrially and conventionally classified in three categories, based on their size and shape: granular carbons, irregular shaped carbons with size ranging from 0.2 to 5 mm [8]; powder carbons, pulverized shaped particles with a predominantly size less than 0.18 mm [9], and carbon fibers [10]. Each type of above-mentioned carbon finds its specific application. For instance, granular carbons are used in liquid and gas phase processes [11]; powder carbons are mainly applied in flue gas treatment [12], and carbon fibers in equipment such as electrodes for electrochemical super-capacitors, hydrogen storage, catalyst support, and so on [13]. Thus, the selection of a proper shape for a specific industrial usage is very important.

Novel carbon structures with specific properties, such as fullerene and carbon nanotubes have been remarkably studied, and their discovery resulted in nanoscience and nanotechnology developments [3]. Carbon spheres and carbon monoliths are also nanostructured materials showing promising results in many fields: supercapacitor [14], gas storage [15], and catalysis supports [16].

Carbon spheres can be produced using a wide range of techniques that lead to differences in term of size, size dispersity, and internal structures. The common route for the synthesis of carbon spheres is the Chemical Vapor Deposition (CVD), in which a gaseous carbon precursor is treated to high temperature in order to induce decomposition, radical formations, and creation of precursor agglomerates [17]. The growth of the agglomerates leads to the development of spheres. Hollow carbon spheres can also be obtained using templates [18]. Another common approach is the hydrothermal carbonization, which is generally used to convert carbohydrates, lignocellulosic materials, and biomasses in carbon at mild conditions in autoclave. Templates can also be added in the precursor solution before the treatment to produce hollow or composite spheres [19]. Recently, the α-cyclodextrins were used as a carbon source in the presence of a hydrophilic non-ionic surfactant, such as Pluronic F127, to produce hollow carbon spheres [20]. Other routes for the preparation of carbon spheres are arc discharge [21], laser ablation [22], and pyrolysis, after the formation of colloidal polymer spheres [23].

Porous carbon monolith is a relatively new material and there is not a clear definition of it. It can be described as a continuous block of carbon wider than 1 mm and with a three-dimensional (3D) shape [18]. It can show different morphologies, such as cylinder, cube, and cuboid, but also a spherical shape. However, it is not easy to prepare a porous carbon monolith. A direct pyrolysis of a carbon precursor generally results in a bulk carbon material with no or not accessible porosity [24]. Thus, a typical approach involves the use of soft or hard templates. In the hard template process, a porous inorganic template is impregnated with a precursor, which is converted in carbon materials, and the template required to be removed by strong acid or base [25]. Many porous inorganic matters can be used as templates, such as zeolite and silica, while glucose and sucrose are generally employed as carbon sources. Alternative to hard template, organic polymers and surfactants have been used as a soft template. Again, they must be removed by thermal decomposition or using suitable solvents [26].

In this paper, a simpler approach to produce carbon spheres with controlled dimensions and carbon monolith that does not require the use of templating agents is presented using as carbon precursor nanosponges based on oligosaccharides. Nanosponges are hyper-crosslinked polymers with a three-dimensional network of cavities and they can be synthesized using a large variety of saccharides, such as building block and cross-linkers [27]. In this paper, two types of nanosponges have been investigated: β-cyclodextrins nanosponges and amylose nanosponges both cross-linked with the pyromellitic dianhydride. In our previous studies [28,29], these two nanosponges have shown to be suitable precursors for the synthesis of microporous carbon materials with a quite high surface area (form 540 to 650 m^2^/g) and narrow pore size distribution (5–16 Å). Herein, we report how to obtain carbon monoliths and carbon microsphere controlling the nanosponge synthesis and the pyrolysis condition, tailoring the final shape and microstructure of the resulting carbon.

## 2. Materials and Methods

### 2.1. Materials

β-cyclodextrin (βCD) and soluble amylose (Linecaps, Lc) were kindly provided by Roquette Italia SPA (Cassano Spinola, Al, Italy). Pyromellitic dianhydride (PMDA), dimethyl sulfoxide (DMSO), acetone, and triethylamine were purchased from Sigma–Aldrich (Merck Life Science S.r.l. Via Monte Rosa, 93, 20149 Milano, Italy).

### 2.2. Synthesis of the βCDNS and LcNS

The β-cyclodextrin nanosponge (βNS) and Linecaps nanosponge (LcNS) were prepared dissolving 11.35 g of anhydrous βCD or Lc and 17.45 g of pyromellitic dianhydride in 100 mL of DMSO containing 2.7 mL triethylamine (19.4 mmol) and were allowed to react at room temperature for 1 h, as described elsewhere [28,29]. Once the reaction is over, the solid obtained could be ground in a mortar and Soxhlet extracted with acetone for 24 h.

### 2.3. Preparation of Carbons

Microsphere preparation: 2 g of βNS or LcNS are put in an alumina combustion boat and then pyrolyzed using a Lenton 1200 tubular furnace. Samples are pyrolyzed with a heating ramp of 10 °C min^−1^ until 800 °C and kept in isotherm for 20 min under a nitrogen flux (65 mL min^−1^). The samples are than cooled to room temperature under nitrogen flux.

Monolith Preparation. The βCD based NS were synthetized dissolving 6.12 g of anhydrous βCD were dissolved in 20 mL of DMSO. After the complete dissolution, 4.50 mL of TEA was added as catalyst. Few minutes later, 4.72 g of pyromellitic dianhydride were mixed and allowed to react at room temperature overnight. Once the reaction was concluded, the block polymer was pulled out. A Soxhlet extractor with acetone was used for the purification. Then the whole block is subjected to the pyrolysis process in the same condition used for the preparation of microsphere. For the LcNS, in a typical procedure, 4.89 g of Lc was solubilized in 20 mL of DMSO in a bottom-rounded flask. Moreover, 3.50 mL of TEA was added and then the 3.76 g of PMDA. After few minutes the cross-linking reaction occurred, and the NS is left to react overnight in order to be considered competed. The LcNS was pulled out and eventually purified in a Soxhlet extractor with acetone for 24 h. The block is then pyrolyzed in a tubular furnace in the same condition used for the preparation of microsphere.

### 2.4. Characterization

Attenuated Total Reflection (ATR) (Perkin-Elmer Spectrum 100) was used for collecting 16 scans for each spectrum of the nanosponges before and after the thermal treatment in the range 4000–650 cm^−1^ at 4 cm^−1^ resolution.

Scanning Electron Microscopy (SEM) (Leica Stereoscan 410) was applied to investigate the nanosponges and the carbons morphology. All of the samples were coated with gold by a sputter coater (Baltec SCD 050) for 60 s under vacuum at a current intensity of 40 mA.

Thermal behavior and stability of the nanosponges were studied by thermogravimetric analysis (TGA) (TGA 2950 balance TA Inc.). Moreover, 15 mg of sample were placed in an alumina crucible and heated to 800 °C with a ramp temperature of 10 °C·min^−1^ under nitrogen flux.

The carbons were characterized by nitrogen adsorption–desorption isotherms at 77 K obtained with an automatic gas-volumetric instrument (ASAP 2010, Micromeritics). The samples were outgassed at 300 °C overnight and analyzed using nitrogen at 77 K. The Langmuir model was applied to evaluate the specific surface area, whereas for the determination of the quantity and dimension of pores, density functional theory (DFT) model were used.

The samples elemental composition was studied using a Thermo Fisher FlashEA 1112 Series elemental analyzer.

## 3. Results and Discussion

**Figure 1:**
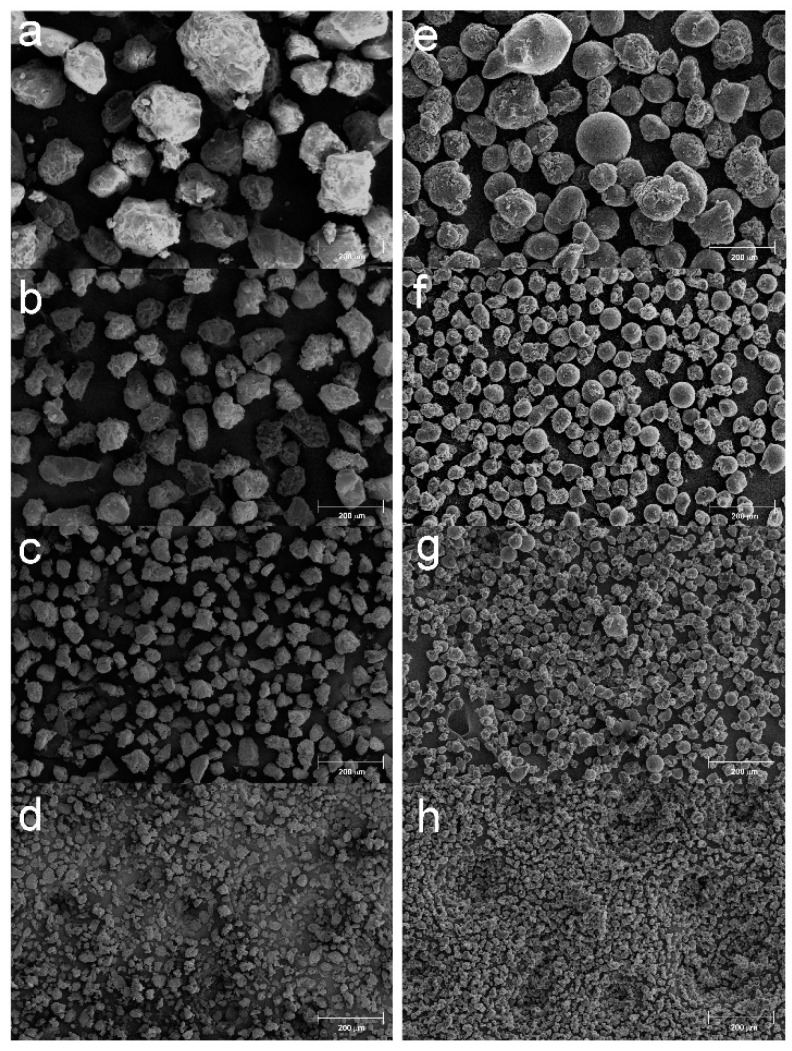
SEM micrographs of β-cyclodextrin nanosponge. (**a**) (βNS) > 100 μm, (**b**) βNS 71–100 μm, (**c**) βNS 40–71 μm, (**d**) βNS < 40 μm, (**e**) C-βNS > 100 μm, (**f**) C-βNS 71–100 μm, (**g**) C-βNS 40–71 μm, (**h**) C-βNS < 40 μm. Magnification: 100 ×.

**Figure 2:**
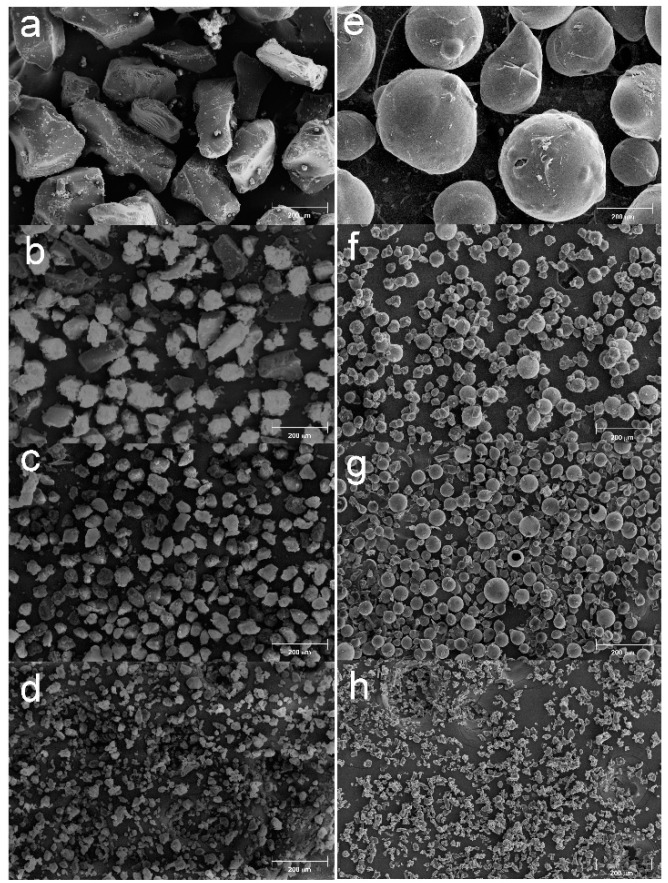
(**a**)SEM micrographs of LcNS > 100 μm, (**b**) Linecaps nanosponge (LcNS) 71–100 μm, (**c**) LcNS 40–71 μm, (**d**) LcNS < 40 μm, (**e**) C-LcNS > 100 μm, (**f**) C-LcNS 71–100 μm, (**g**) C-LcNS 40–71 μm, (**h**) C-LcNS < 40 μm. Magnification: 100 ×.

The βNS and the LcNS are synthesized with the method previously described. The synthesis of both the samples leads to the formation of a solid block that can be ground before being purified in Soxhlet. In our previous works [28,29], it has been reported that from pyrolysis of powdered nanosponges it is possible to obtain microporous carbons with spherical morphology. In order to control the dimension and the formation of carbon sphere, the nanosponges were ground and sieved in four parts with different particle sizes: higher than 100 μm, between 100 and 71 μm, between 71 and 40 μm, and less than 40 μm. These four portions were pyrolyzed in a tubular furnace at 800 °C in inert atmosphere. Figure 1 shows the micrograph collected for the carbons from βNS (C-βNS) and Figure 2 depicts the carbons from Lc-NS (C-LcNS) derived from the different granulometric fractions of βNS and LcNS, respectively. For comparison, the several batches of sieved nanosponges are also reported in Figure 1 and in Figure 2 in order to confirm the granulometric separation and to study the dependence of the spherical shape on the initial particle size.

Regarding the bare βNS (Figure 1a–d), it can be observed that all the particles showed an irregular polyhedral morphology, mainly due to the grinding process, but the dimensional separation successfully occurred. The carbon from βNS (Figure 1, panel e) displays the ability to produce a spherical morphology for NS particles bigger than 100 μm. Reducing the size of the starting particles (71–100 μm—panel f), the morphology of the carbon particles became more irregular, but the spherical morphology is still predominant. Referring to panel g, which shows the carbons form NS grounded between 71 and 40 μm, it can be notice that the spherical shape has almost disappeared, but there are still some spheres. The sphere morphology disappears pyrolyzing the βNS below 40 μm (Figure 1, panel h).

**Figure 3:**
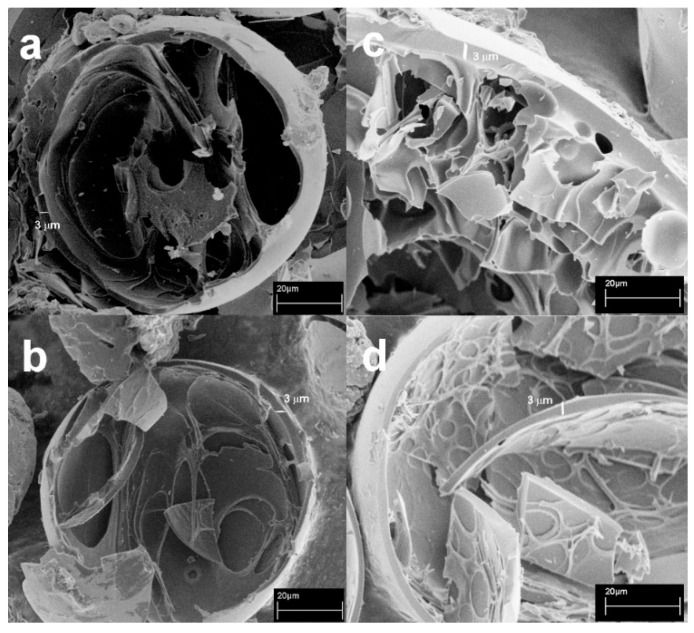
(**a**) SEM micrographs of C-βNS > 100 μm, (**c**) C-βNS 71–100 μm. Magnification: 700 ×; (**b**) C-LcNS > 100 μm, (**d**) C-LcNS 71–100 μm. Magnification: 800 ×.

In Figure 2 are reported the SEM image of the NS obtained from Line Caps and the respective carbon particles. The bare LcNS (Figure 2a–d) shows particles with polyhedral morphology, but the dimensional separation successfully occurred. As seen for the carbons from βNS, a spherical morphology has been obtained starting form LcNS with particle size wider than 100 μm (panel e). Decreasing the dimension of the particles (71–100 μm—panel f), the morphology of the sample remains mainly spherical. Further decreasing the particles size (40–71 μm—panel g), the presence of spherical shape begins to be no longer the predominant one and it disappears for the carbon from LcNS with particle size of 40 μm (panel h). Thus, the influence of the granulometry on the morphology of the resultant carbons is related with the size of the starting materials. The spheres are obtained when a nanosponge, with size up to 71 μm, is pyrolyzed, and disappears when NS with dimension inferior to 40 μm are used. To see the inner part of the spheres, some of them were broken, and are shown in Figure 3. As can be seen, the spheres are hollow and formed of a carbon shell, whose thickness seems to remain constant despite the variation of the size of the starting precursor particle.

The formation of this morphology can be related with the degradation pathway followed by the hyper-crosslinked polymer during the pyrolysis.

During the heat treatment, the nanosponge softens passing through a fluid phase while pyrolysis is taking place. The surface tension of the fluids leads to the formation of spherical particles. Due to the temperature gradient between the outside and inside of the spheres, the carbonization begins on the surface, this causes the formation of a solid carbon shell, while the carbonization continues from the outside towards the inside of the still fluid particle. The particle should be reduced in size since the pyrolysis process causes the volatilization of at least the 60% by weight of the polymer, but this is not possible since the outer shell is solidified. The volume reduction then occurs at the expense of the still fluid internal mass, which withdraws towards the area already carbonized, leaving a vacuum inside the particle.

**Figure 4:**
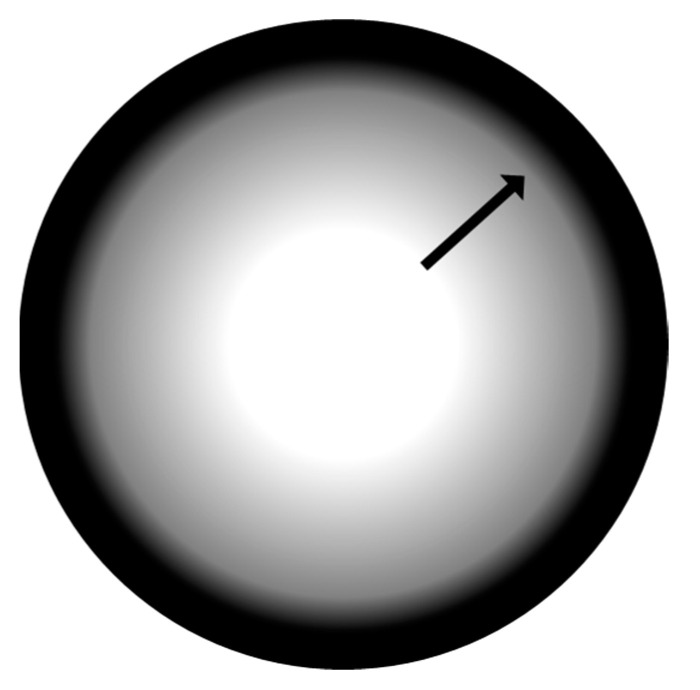
Representative sphere formation scheme; the arrow indicates the direction of contraction of the part still soft towards the solid outer shell.

Figure 4 shows a scheme of this process. The carbon shell reaches a thickness of about 3 μm, meaning that there is a limit for the formation of particles with spherical shape. Precursor particles smaller than 40 μm do not allow the creation of a temperature gradient enough to allow this mechanism and below this value; the thickness of the surface coincides with the overall dimension of the particles not allowing to assume the spherical morphology, and so it remains with the same shape of the starting materials.

**Figure 5:**
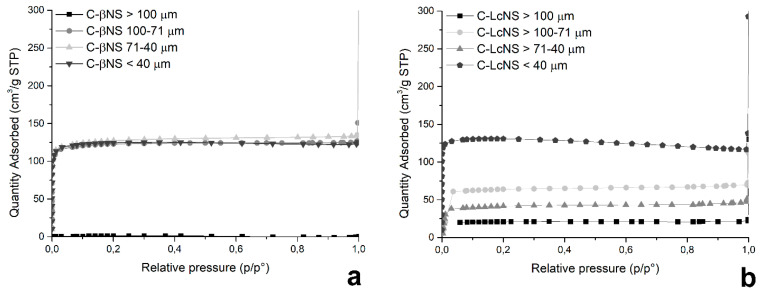
Isotherms of C-βNS (**a**) and C-LcNS (**b**).

The samples of C-βNS and C-LcNS were analyzed with the ASAP 2010 to evaluate their physical properties. The objective of this measure is to the determinate the dependence between the granulometry of the NS and the development of the specific surface area of the resulting carbons. In Figure 5, all of the collected isotherms for C-βNS (a) and C-LCNS (b) are reported, and all of the results are listed in Table 1 and Table 2.

The trend of the adsorption–desorption isotherms shows that the pyrolysis generates microporous carbons. No capillary condensation phenomena can be observed. Indeed, isotherms recorded are in accordance with Type I isotherm and, following the International Union of Pure and Applied Chemistry (IUPAC) classification, characteristic of microporous samples. The Type 1 isotherm can be described by the Langmuir equation, which was developed assuming the homogeneity and mono-layered adsorption on the surface. From the results listed in Table 1, it can be seen that the specific surface area of the spheres from C-βNS is around 550 m^2^/g and the porosity and the dimension of pores is the same for all the samples having dimensions less than 100 μm. For the C-βCD with dimensions higher than 100 μm, the recorded area is lower than for the other carbons, around 7 m^2^/g. Therefore, below 100 μm, the granulometry of the βNS has no influence on the specific surface area, and in the resulting porosity of the final carbons. Regarding the carbons from LcNS, when the size of the NS decreases, there is a progressive development of the specific surface area. It passes from 93 m^2^/g for the C-LcNS with dimension higher than 100 μm, to 573 m^2^/g for the carbon from LcNS sieved below 40 μm. The volume of pores also increases as the size of the particles are reduced, whereas their width remains constant. Therefore, for LcNS there is a strong dependence of the granulometry of the starting materials on the physical characteristics of the resulting carbons. The effect of particle size is a complex phenomenon and it is often associated with change in physical properties of samples. It is possible to suppose that the different structure of the native βNS and LcNS have an influence on reorganization reactions that contribute to the specific surface area. Indeed, the building block of the βNS are the β-cyclodextrins that are cyclic oligosaccharides composed by seven glucopyranose units linked by α-(1-4) bonds. The building block of the LcNS are the Linecaps. Similarly to the cyclodextrins, they are a starch derivative, and they are polymeric carbohydrates consisting in anhydroglucose units linked together primarily through α-(1-4)-glucosidic bonds, but they contains two microstructures. The first is amylose, a linear structure of α-1,4 linked glucose units. The second is the amylopectin, a high branched structure composed of short α-1,4 chains linked by α-1,6 bonds. The Linecaps have a helical design with an outer hydrophilic surface, whereas the inner surface is hydrophobic. This different structure of the bare samples could be responsible of the reorganization reactions that occur during the pyrolysis and lead to a diverse evolution of the specific surface area.

**Figure 6:**
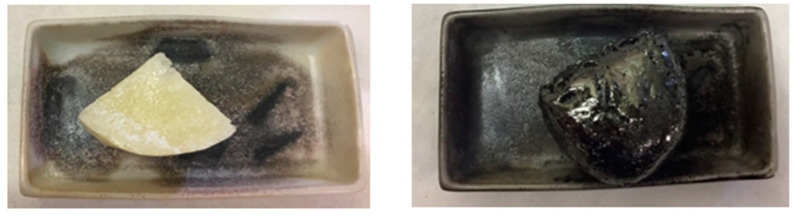
Portion of βNS before and after the pyrolysis.

Having found out that it is possible to modulate the specific surface area and the morphology of the resulting carbon from βNS and LcNS, tuning the dimension of the particles, the possibility to obtain monolithic carbons was explored. After the cross-linking reaction between the βCD or Lc with the pyromellitic dianhydride, the products obtained are solid blocks (see Supplementary Materials). A part of the two blocks of βNS and LcNS was pyrolyzed in a tubular furnace to obtain carbons with the same morphology of the starting materials. Figure 6 shows a piece of βNS before and after the pyrolysis.

As shown in the Figure 6, the shape of the nanosponge remains unchanged after the pyrolysis process. Thus, it suggests that it is possible to make carbon with different dimensions and shapes.

**Figure 7:**
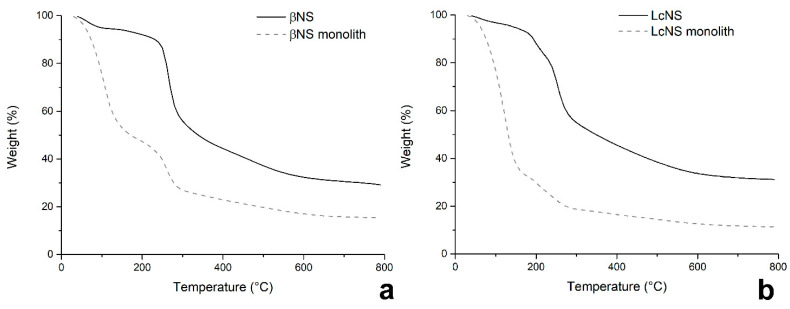
TGA of βNS (**a**) and LcNS (**b**).

As a first step, the possibility to obtain monolithic carbons by direct pyrolysis of the whole block of NS was evaluated, without any further purification usually performed after the synthesis. The NS blocks were analyzed using TGA; the thermograms of βNS, LcNS, and of the respective powdered and purified nanosponges are reported in Figure 7.

The thermograms of the βNS (a) shows three steps of weight loss, which occur at the same temperature. The thermal degradation of the βNS purified (solid black line Figure 7a) has a first step of weight loss that occurs before 180 °C with a 7% of weight loss which is related with the moisture adsorbed by the sample. A second step takes place between 220 °C and 350 °C with a 42% of weight loss. The last one goes from 350 °C to 800 °C losing a 14% of its mass. The carbon yield of the pyrolysis is 28%. The thermogram of βNS not purified shows a first step of weight loss until 180 °C, which causes a 51% of weight loss. During this phase, it is possible to suppose that the volatiles released at this temperature are either water adsorbed, but also by-products and solvent (DMSO). Indeed, taking as 100% the weight of the sample at 180 °C, the char yield at 800 °C is 28% as the pyrolysis yield of the βNS purified.

The thermogram of the LcNS purified (solid black line, Figure 7b) show three steps of weight loss as the βNS. The first one occurs before 180 °C; it belongs to the evaporation of the moisture and it corresponds to 5%. The second step goes from 160 °C to 310 °C with a 51% of weight loss. The last stage takes place between 310 °C and 600 °C. The pyrolysis yield in this case is 32%. As for the βNS, it is possible to see that the LcNS not purified (dotted grey line, Figure 7b) shows an initial weight loss of solvent and other by-products before 180 °C.

The presence of DMSO in the monoliths is confirmed by the CHNS analysis (Table 3). In the case of non-purified NS, a huge amount of sulfur, around 20 wt. %, is still present.

The physical properties of the not purified NS are listed in Table 4. As can be seen, the monolithic carbon obtained from βNS has a low specific surface area and porosity, while in the case of that obtained from LcNS it was not possible to detect the specific surface area.

In order to improve the physical characteristic of the monolithic carbons, the solid blocks obtained after the synthesis of βNS and LcNS were purified in Soxhlet with acetone. The trend of purification was checked with CHNS analysis. The results are reported in Table 5.

The target of the purification is the elimination the residual DMSO and it is possible to see that 18 h of Soxhlet extraction are not enough to completely remove the solvent. The complete elimination of the DMSO was reached after 27 h of Soxhlet extraction.

**Figure 8:**
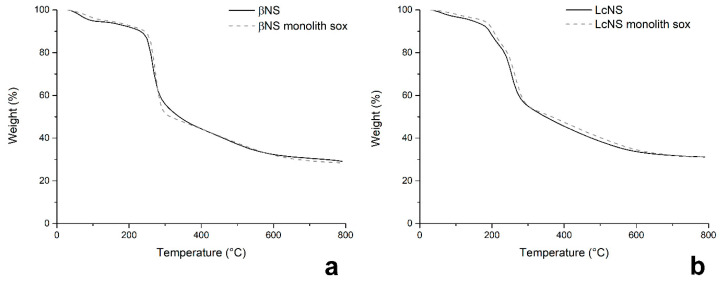
TGA of βNS (**a**) and LcNS (**b**).

This is confirmed by TGA (Figure 8) where the thermograms of the βNS and LcNS block and βNS and LcNS crushed are perfectly overlapped; hence, the purification step worked successfully. The monolithic NS were then pyrolyzed and the respective monolithic carbons were analyzed for checking the specific surface area and the porosity. All of the data recorded are summarized in Table 6.

The shape of the isotherms for all the samples is a Type 1, so they are microporous materials with a high specific surface area (610 m^2^/g for C-βNS and 460 m^2^/g for C-LcNS) with a narrow pore size distribution (5–11 Å for C-βNS and 5–15 Å for C-LcNS) comparable with that obtained for the hollow carbon microspheres.

## 4. Conclusions

In the present work, hollow carbon microspheres and carbon monolith were successfully prepared via carbonization of hyper-crosslinked polymer based on either cyclodextrins or amylose in a template-free way. In particular, it is possible to obtain microporous carbon monoliths by simply pyrolyzing the nanosponge blocks after the hyper-crosslinked polymer synthesis. The purification steps seem to play an important role in the development of the surface area, up to 610 m^2^/g. Moreover, grinding the nanosponges, it is possible to have carbon microspheres with high specific surface area and a narrow pore size distribution. The possibility to tailor the shape of the carbons makes the nanosponge a suitable precursor for different industrial applications.

## Figures and Tables

**Table 1:** Specific surface area, cumulative pore volume and pore width of the C-βNS.

Sample	Specific Surface Area (m^2^/g)	Cumulative Pore Volume (cm^3^/g)	Pore Width (Å)
C-βNS > 100 μm	7	0.001	14–24
C-βNS 71–100 μm	565	0.143	6–15
C-βNS 40–71 μm	544	0.141	5–17
C-βNS < 40 μm	550	0.118	6–13

**Table 2:** Specific surface area, cumulative pore volume and pore width of the C-LcNS.

Sample	Specific Surface Area (m^2^/g)	Cumulative Pore Volume (cm^3^/g)	Pore Width (Å)
C-LcNS > 100 μm	93	0.028	16–21
C- LcNS 71–100 μm	189	0.056	12–18
C- LcNS 40–71 μm	284	0.089	13–18
C- LcNS < 40 μm	573	0.129	5–15

**Table 3:** CHNS of the βNS and LcNS, ground and purified, and βNS and LcNS monoliths without purification.

Sample	%C	%H	%N	%S
βNS	51.32	6.50	2.86	0.00
βNS monolith	34.61	6.88	1.33	19.46
LcNS	49.56	6.53	3.01	0.00
LcNS monolith	32.99	6.86	1.24	20.33

**Table 4:** Specific surface area, cumulative pore volume, and pore width of the C-βNS C-LcNS with no purification after the synthesis.

Sample	Specific Surface Area (m^2^/g)	Cumulative Pore Volume (cm^3^/g)	Pore Width (Å)
C-βNS monolith	138	0.010	13–17
C-LcNS monolith	/	/	/

**Table 5:** CHNS results.

Sample	%C	%H	%N	%S
βNS monolith 0 h Soxhlet	34.61	6.88	1.33	19.49
βNS monolith 18 h Soxhlet	50.15	6.38	2.95	0.14
βNS monolith 27 h Soxhlet	49.99	6.69	3.29	0.00
LcNS monolith 0 h Soxhlet	32.99	6.86	1.24	20.33
LcNS monolith 18 h Soxhlet	49.30	6.41	3.12	0.04
LcNS monolith 27 h Soxhlet	50.05	6.64	3.16	0.00

**Table 6:** Specific surface area, cumulative pore volume, and pore width of the C-βNS.

Sample	Specific Surface Area (m^2^/g)	Cumulative Pore Volume (cm^3^/g)	Pore Width (Å)
C-βNS monolith sox	610	0.174	5–11
C-LcNS monolith sox	460	0.114	5–15

## Supplementary Materials

The following are available online, Figure S1: FT-IR Spectra of βNS monolith before and after 18 and 27 h of extraction, Figure S2: FT-IR Spectra of LcNS monolith before and after 18 and 27 h of extraction, Figure S3: a) carbon from a-CD-NS 100 ×; b) the inner part of a-CD-NS carbon 500 ×; c) carbon from g-CD-NS 100 ×; d) the inner part of g-CD-NS carbon 500 ×, Figure S4: SEM image of the carbons form b-CD cross-linked with the pyromellitic dianhydride in molar ratio 1:2.
