# Supplementary figures and images for: Preparation of Microspheres and Monolithic Microporous Carbons from the Pyrolysis of Template-Free Hyper-Crosslinked Oligosaccharides Polymer
Source: Molecules. 2020 Jul 2;25(13):3034. doi: 10.3390/molecules25133034 (PMC7411619; doi:10.3390/molecules25133034)

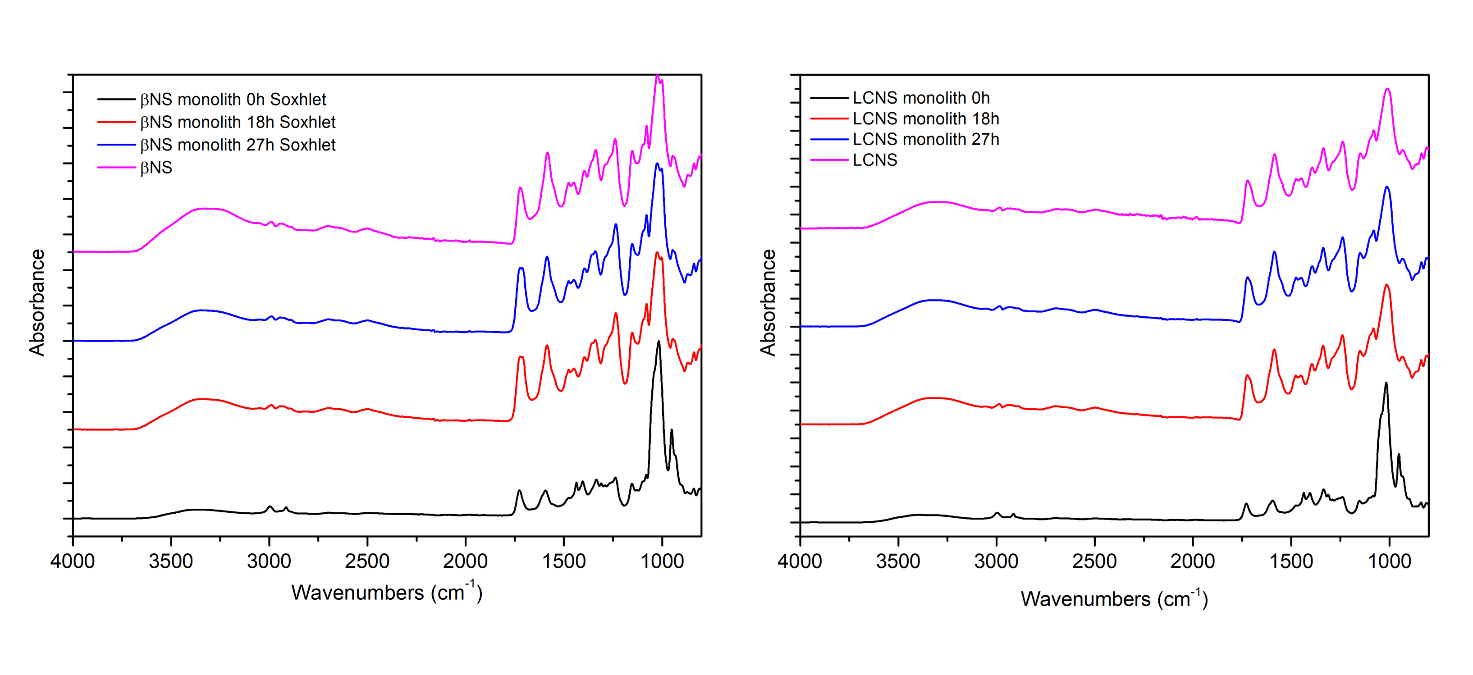

Supplement: Supplementary file 1 [file molecules-25-03034-s001.zip › Figure S1.tif]

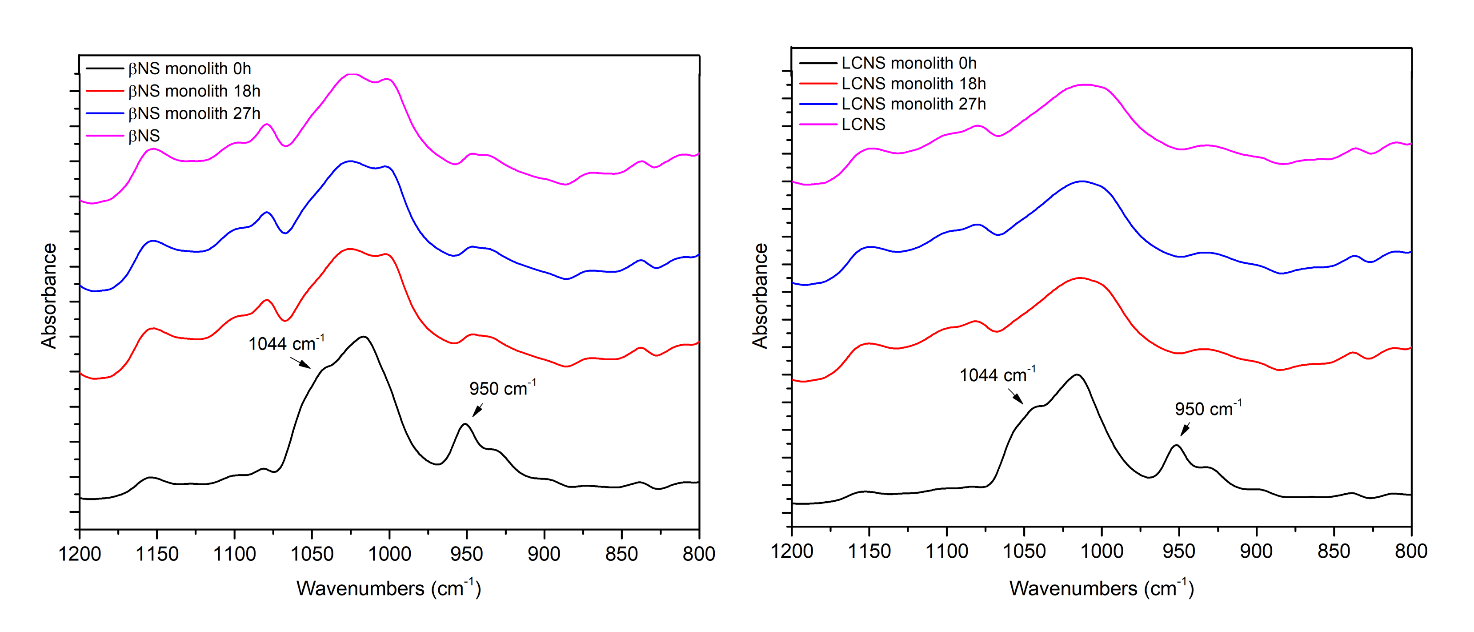

Supplement: Supplementary file 1 [file molecules-25-03034-s001.zip › Figure S2.tif]

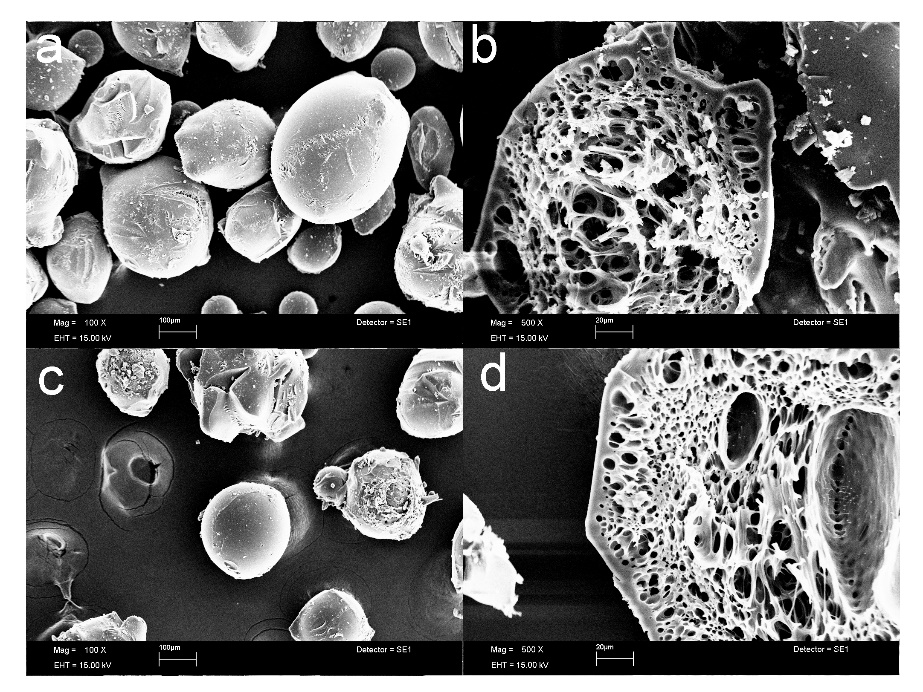

Supplement: Supplementary file 1 [file molecules-25-03034-s001.zip › Figure S3.tif]

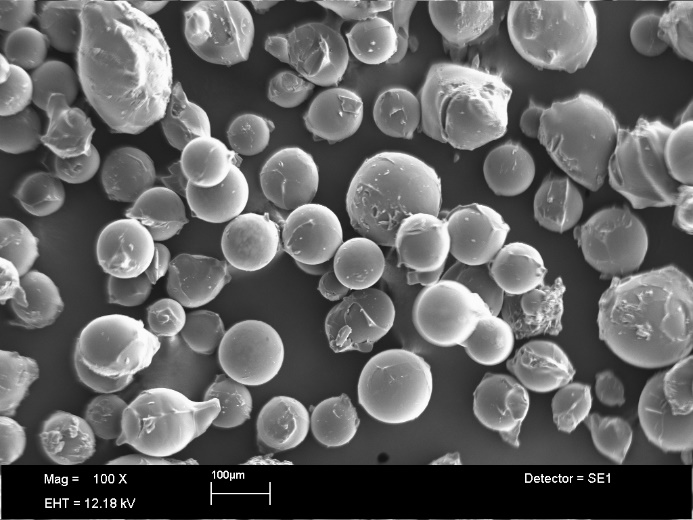

Supplement: Supplementary file 1 [file molecules-25-03034-s001.zip › Figure S4.tif]
